# Hodgkin lymphoma in adolescent and young adults: insights from an adult tertiary single-center cohort of 349 patients

**DOI:** 10.18632/oncotarget.20684

**Published:** 2017-09-06

**Authors:** Camille Bigenwald, Jacques-Emmanuel Galimard, Laurent Quero, Aurélie Cabannes-Hamy, Catherine Thieblemont, Nicolas Boissel, Pauline Brice

**Affiliations:** ^1^ Department of Onco-Hematology, Hôpital Saint Louis, Assistance Publique des Hôpitaux de Paris (AP-HP), Université Paris, Paris, France; ^2^ Department of Biostatistics and Medical Information (ECSTRA Team), Center of Research in Epidemiology and Statistics Sorbonne, Inserm UMR 1153, Université Paris Diderot, Paris, France; ^3^ Department of Radiation Oncology, Hôpital Saint Louis, AP-HP, Université Paris, Paris, France; ^4^ Hematology Adolescents and Young Adults Unit, Hôpital Saint-Louis, AP-HP, Université Paris, Paris, France

**Keywords:** Hodgkin, young adults, adolescents

## Abstract

**Background:**

Adolescent and young adults (AYA) represent one third of patients affected by Hodgkin lymphoma (HL). These patients are frequently treated either with pediatric or adult protocol depending on their physician background. This population has been understudied so far, in terms of HL characteristics and treatment-associated outcomes.

**Aim:**

We aimed to extensively describe HL features in the AYA population including HL characteristics, progression-free (PFS) and overall survival (OS).

**Methods:**

From 1979 to 2013, consecutive patients with HL aged between 15 to 25 years and followed at Saint-Louis Hospital were prospectively enrolled. Survivals were estimated using the Kaplan-Meier method.

**Results:**

349 patients were included and studied, with a median follow-up of 7 years. The majority of patients were treated with adult protocols (mainly ABVD and BEACOPP). They presented adverse clinical characteristics with a high proportion of stage III and IV according to Ann Arbor classification (45 %), a high rate of B symptoms (46 %) and extra-nodal involvement (36 %). Despite these pejorative clinical features, the prognosis remains good with a 10-year PFS and OS estimated at 81.0 % (95%CI [76.7-85.5]) and 90.7% (95%CI [87.2-94.4]), respectively. In multivariate analysis, stages III and IV according to Ann Arbor classification, mixed cellularity histology, elevated neutrophils and LDH above range were independently associated with a worse PFS. We identified a subgroup of 11 primary refractory patients with a particularly poor prognosis. The toxicity rate was low (7.4 %).

**Conclusion:**

Despite their baseline pejorative features, AYA with HL have a good prognosis. Progresses are still needed in order to reduce toxicities. Primary refractory patients with a particularly poor prognosis should be detected early in order to quickly introduce new targeted therapies.

## INTRODUCTION

Classical Hodgkin lymphoma (HL) is one of the most common malignancy in adolescent and young adults (AYA) [1]. About 30 % of HL cases occur in patients aged from 15 to 25 years old [2]. AYA represent a challenging group of patients in terms of diagnosis and treatment. Delayed diagnoses have been observed in this specific population and treatments’ side effects can lead to psychological disturbances in young patients [3, 4]. Moreover, there is no established standard of care for AYA’s HL and it is worth noticed that AYA patients are frequently excluded from clinical trials [5]. In order to ensure an optimal medical and psychological care in this population, dedicated units and/or programs have recently been implemented in many countries. Those patients are currently treated either with pediatric or adult protocols depending on local clinical pathways and mainly relying on physician background. Both approaches have been shown to be effective, with excellent remission and overall survival rates [6]. However, no prospective trial comparing pediatric and adult treatment in this population has been conducted to date.

Most of the literature includes AYA patients with HL into either pediatric or adult population, instead of considering this subgroup of patients as a distinct entity. This leads to a limited knowledge regarding HL specific characteristics in AYA. Thus, little is known about epidemiology, everyday life practice and outcome of this particular group of patients. Therefore, from a tertiary single-center cohort of 349 AYA patients with HL, we conducted a study that aimed to investigate HL specificities in AYA.

## RESULTS

### Patients’ baseline characteristics

A total of 349 patients aged between 15 and 25 years old with a histological confirmation of classical HL were enrolled. Baseline characteristics are displayed in Table 1. None of the patients were HIV positive. The median age at diagnosis was 21 years. There were slightly more female (N= 201, 57.6 %). Of note, although the majority of patients presented a localized disease, disseminated disease i.e. stage III and IV according to Ann Arbor classification was over represented (N= 156, 44.7 %). Ninety-seven patients (28.2 %) had a bulky disease, 115 patients (35.7 %) presented an extra nodal involvement and lung was the most frequently involved organ (N= 59, 17.4 %).

**Table 1:** Patient characteristics at diagnosis, n= 349

	N (%) or median [IQR]	Available for
**General characteristics**		
Female	201 (57.6 %)	349
Age at HL diagnosis (years)	21 [19.2-23.0]	349
Ann Arbor stage		349
Stage I	40 (11.5 %)	
Stage II	153 (43.8 %)	
Stage III	54 (15.5 %)	
Stage IV	102 (29.2 %)	
B symptoms	149 (45.9 %)	324
IPS for stage III-IV	2 [2-3]	126/156
ECOG performance status		339
0	269 (79.4 %)	
1	64 (18.8 %)	
2	5 (1.5 %)	
3	1 (0.3 %)	
Histologic subtype		347
Nodular sclerosis	300 (86.5 %)	
Mixed cellularity	30 (8.6 %)	
Lymphocyte predominance	5 (1.4 %)	
Lymphocyte depletion	0	
Unclassified	12 (3.5 %)	
Negative EBV-LMP staining	128 (83.7 %)	153
**Nodal involvement**		
Cervical nodes	294 (85.2 %)	345
Axillary nodes	75 (22.1 %)	340
Mediastinal nodes (including Bulky disease)		344
Absent	55 (16.0%)	
Present	192 (55.8%)	
Bulky disease	97 (28.2%)	
Aortic nodes	84 (24.7 %)	340
Iliac nodes	12 (3.6 %)	338
Mesenteric nodes	26 (7.7 %)	336
Pelvic nodes	27 (8.0 %)	337
Spleen	58 (17.1 %)	340
**Extra-nodal involvement**		
Extra-nodal involvement	115 (35.7 %)	322
Lung	59 (17.4 %)	340
Pericardia	27 (8 %)	338
Bone	26 (8 %)	326
Pleura	26 (7.7 %)	339
Liver	10 (2.9 %)	339
Bone marrow	7 (2.1 %)	332
Oro-pharyngeal	3 (0.9 %)	338
Epiduritis	3 (0.9 %)	339
**Biological characteristics**		
Hemoglobin (g/dl)	12 [11-13.5]	314
Leukocytes (G/l)	10.65 [8.1-13.9]	288
Albumin (g/dl)	4 [3.7-4.4]	209
Lymphocytes (% of white cell count)	15 [11-21]	265
Neutrophils (% of white cell count)	75 [70-80]	267
Platelets	372.5 [289.8-445.0]	232
Lactate dehydrogenase (above normal range)	80 (30.7 %)	261
Erythrocyte sedimentation rate (mm/hour)	53 [26-77]	321

IPS: International Prognostic Score, ECOG, Eastern Cooperative Oncology Group, EBV-LMP: Epstein-Barr Virus Latent Membrane Protein.

As in the literature, the AYA population may be divided into patients aged less than 21 years and patients aged 21 and over [9], we compared the main characteristics between these 2 groups (Supplementary Table 1). No difference was noticed in terms of histologic subtype, Ann Arbor stage, performance status and LDH. Patients less than 21-year-old tended to have a HL with a less frequent association with EBV (p= 0.049).

We further compared the HL population diagnosed before and after June 2005, corresponding to the date of the advent of PET-CT (Supplementary Table 2). To note, patients presented more frequently a disseminated disease which corresponds to stage III and IV according to Ann Arbor classification (40.5 % versus 52.0 %, p= 0.04), when the diagnosis was made after June 2005. Conversely, the rate of cervical and axillary involvement was lower in patients diagnosed after June 2005 (89.0 % versus 78.7%, p= 0.01; and 28.2 % versus 11.8%, p< 0.01, respectively).

### Treatment

All patients received chemotherapy. 177 patients (50.7 %) have been included in an academic therapeutic clinical trial (described in Supplementary Table 3). First line treatment characteristics are summarized in Supplementary Table 4. ABVD was the most frequently used regimen (60.2 %). BEACOPP has been used in 53 patients (15.2 %). 204 patients (58.5 %) underwent a complementary radiotherapy. 80.4 % of the patients who underwent a RT had a localized disease (stage I or II according to Ann Arbor classification). The median dose was 36 Gy. Complete remission was achieved in 338 patients (96.8 %) after the first line treatment.

### Management at first relapse/progression

Fifty-eight events other than death occurred during follow-up, including 11 primary refractory patients and 47 patients who relapsed after the first line treatment (Supplementary Table 5). The median time to relapse was 0.86 year (IQR [0.40 – 1.75]). Forty-seven patients, including primary refractory and relapsed patients, underwent autologous stem cell transplant (ASCT). Among them, 37 were performed after one salvage chemotherapy regimen and 10 after 2 or more salvage chemotherapies to achieve remission. The patients who did not undergo an ASCT either declined the treatment (n= 3), never obtained a complete remission (n= 2) or presented a late and localized relapse (n= 6) so their physicians decided to deny the ASCT indication. Those 6 patients with late relapse never presented a second relapse.

The most commonly used salvage chemotherapy regimens were MINE (mitoguazone, ifosfamide, vinorelbine, etoposide) and IVOx (ifosfamide-etoposide, oxaliplatin) [10, 11]. The most frequently used intensification regimen was BEAM in 40 out of 47 patients. The 7 patients who were not intensified with BEAM, were conditioned with CBV (n= 6) or BCNU-Endoxan-VP16 (n= 1) [12]. Allogeneic stem cell transplantation was performed in 7 patients who presented either a relapse after an auto-SCT (n= 4) or an early and disseminated relapse (n= 3). For the latter, they received a tandem auto-SCT – allo-SCT as recommended by the Lymphoma Study Association (LYSA) [8]. The 7 allo-SCT were performed either with siblings or matched unrelated donors.

### Comparison of refractory and relapsed patients

The clinical characteristics at diagnosis between the 11 refractory and the 47 relapsed patients are summarized in Table 2. While non-statistically significant, the refractory patients seem to present more frequently a bulky mediastinum compared to the relapsed patients (54.6 % versus 23.4 %). Among the refractory patients, 10 patients underwent an autologous stem cell transplantation and they all relapsed after the ASCT. None underwent an allogeneic stem cell transplantation due to chemoresistance. The estimated overall survival at 5 years for refractory patients was 21.2 % (95%IC [6.3-71.6]) compared to 75.3 % (95%CI [66.7-91.0]) for relapsed patients (p<0.01).

**Table 2:** Comparison between refractory patients and relapsed patients

HL characteristics at diagnosis	Record for	Refractory patients, n= 11	Relapsed patients, n= 47	p
**General characteristics**				
Female, n (%)	58	6 (54.6 %)	23 (49.0 %)	1.00
Age at HL diagnosis (years), median [IQR]	58	20.9 [19.6 – 22.6]	21.1 [18.9 – 23.1]	0.97
Ann Arbor stage, n (%)	58			0.51
Stages I and II		3 (27.3 %)	19 (40.4 %)	
Stages III and IV		8 (72.7 %)	28 (59.6 %)	
ECOG performance status, n (%)	55			0.43
0		6 (60 %)	34 (75.6 %)	
>= 1		4 (40 %)	11 (24.4 %)	
Histologic subtype, n (%)	58			0.41
Nodular sclerosis		8 (72.7 %)	35 (74.5 %)	
Mixed cellularity		2 (18.2 %)	11 (23.4 %)	
Lymphocyte predominance		0	1 (2.1 %)	
Unclassified		1 (9.1 %)	0	
LMP staining, n (%)	28			1.00
Positive		0	4 (16 %)	
Negative		3 (100 %)	21 (84 %)	
**Nodal involvement**				
Mediastinal involvement, n (%)	58			0.11
Absent		0	4 (8.9 %)	
Present		5 (45.4 %)	32 (68.0 %)	
Bulky disease		6 (54.6 %)	11 (23.4 %)	
**Extra-nodal involvement**				
Extra-nodal involvement	54	4 (44.4 %)	18 (40 %)	1.00
Lung	57	3 (27.3 %)	12 (26.1 %)	1.00
Pericardia	55	1 (10.0 %)	4 (8.9 %)	1.00
Pleura	56	0	7 (15.6 %)	0.32
**Biological characteristics**				
Hemoglobin (g/dl), median [IQR]	40	11.3 [10.2 – 12.3]	12.3 [11.1 – 13.0]	0.24
Leukocytes (G/l), median [IQR]	34	17.3 [10.2 – 29.5]	11.0 [9.5 – 14.9]	0.20
Albumin (g/dl), median [IQR]	25	38.0 [34.0 – 38.0]	38.5 [31.8 – 43.0]	0.71
Lymphocytes (% of white cell count), median [IQR]	30	10.0 [8.3 – 12.5]	12.5 [9.5 – 15.0]	0.48
Lactate dehydrogenase (above normal range), %	35	2 (33.3 %)	15 (51.7 %)	0.66
Autologous stem cell transplantation, n (%)	57	10 (90.9 %)	37 (78.7 %)	
Double		3 (27.3 %)	4 (8.5 %)	
Allogeneic stem cell transplantation, n (%)		0	7 (14.9 %)	
Overall survival at 5 years, % [95%CI]	58	21.2 % [6.3-71.6]	77.9 % [66.7-91.0]	**<0.01§**

§ log rank test.

### PFS, OS, long-term toxicities and factors associated with the occurrence of an event

At the end of follow-up (median follow-up of 6.9 years (IQR: [4.6; 10.9]), 314 patients (90 %) had achieved a complete remission (CR) or uncertained CR (CRu) [13], 6 patients (1.7 %) were still under treatment for a refractory disease or a relapse and 29 patients died (8.3 %). Details are provided in Table 3. Among the whole cohort, 26 patients (7.4 %) presented a severe treatment-related toxicity. The toxicities observed were 12 solid tumors in 12 patients (5 breast cancers, 2 schwannomas, 1 neurofibrosarcoma, 1 lung cancer, 1 cervical cancer, 1 osteosarcoma, 1 craniopharyngioma), 4 secondary acute myeloid leukemias, 5 heart failures, 2 radiation induced pleuropericardia, and 1 septic shock. Among these patients, 6 received at least 2 regimens of chemotherapy, thus 10.3% of relapsed patients presented a treatment-related toxicity, and 21 received radiotherapy. The cumulative incidence of mortality and especially the treatment- related mortality are displayed in Supplementary Figure 1.

**Table 3:** Patients outcomes

**Outcome, n (%)**	
Death	29 (8.3 %)
Complete remission after 1^st^ regimen	283 (81.1 %)
Complete remission after 2^nd^ regimen	29 (8.3 %)
Late complete remission	2 (0.6 %)
Currently under treatment	6 (1.7 %)
**Cause of deaths, n (%)**	
Hodgkin lymphoma	21 (72.4 %)
Treatment related toxicity	7 (24.1 %)
Other cause	1 (3.5 %)

**Figure 1:**
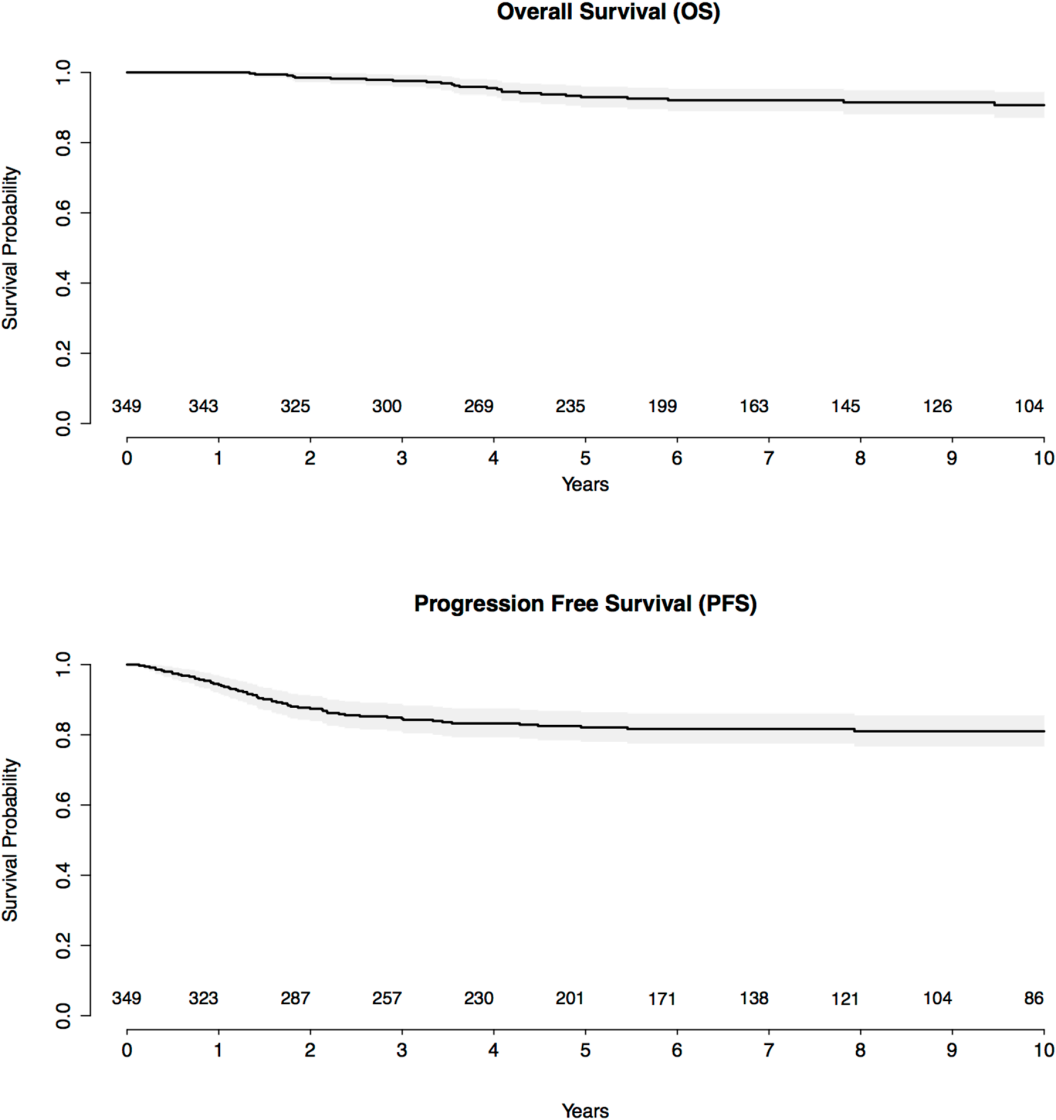
Kaplan-Meier survival curves for progression-free survival and overall survival, with number of subjects at risk and 95% confidence limits

The 5-year PFS and OS estimates were 82.1 % (95%CI [78.0-86.4]) and 93.0% (95%CI [90.1-95.9]) respectively. The 10-year PFS and OS estimates for the whole cohort were 81.0 % (95%CI [76.7-85.5]) and 90.7% (95%CI [87.2-94.4]), respectively (Figure 1). Comparisons of 10-year PFS and OS between patients with localized vs. disseminated diseases, bulky vs. non bulky mediastinum, < 21-year old vs. > 21-year old and diagnoses before and after 1995 and between the type and the dose of radiotherapy are displayed in Supplementary Figure 2. In terms of treatment, MOPP-like treatment and mantle-field radiation were associated with a worse PFS than the other types of chemotherapy and radiotherapy (HR= 2.17 [1.23 - 3.82], p< 0.01 and HR= 10.51 [3.69 - 29.95], p<0.01 respectively) as described in Supplementary Table 6. Moreover, 20 Gy radiation was associated with a worse PFS (p<0.01), whereas 30 Gy radiation was associated with the best PFS among the different radiation doses (Supplementary Figure 2).

In multivariate analysis, stages III and IV according to Ann Arbor classification (HR= 2.09 [1.22 - 3.60], p< 0.01), mixed cellularity histology (HR= 2.62 [1.31 - 5.23], p< 0.01), elevated neutrophils (HR= 1.66 [1.10 - 2.51], p= 0.02) and LDH above range (HR= 1.94 [1.07 - 3.51], p= 0.03) were independently associated with a worse PFS (Supplementary Table 6).

## DISCUSSION

Here we described a large cohort of 349 AYA with HL followed in a single tertiary academic center and consecutively enrolled from 1979 to 2013. Our study showed that AYAs with HL present severe clinical features at diagnosis (45 % of disseminated disease, 46 % of B symptoms, 28 % of bulky disease, 36 % of extra-nodal involvement) and a very high frequency of mediastinal involvement (84 %). Conversely, it has been previously described that children with HL are classically diagnosed with a localized disease [14, 15] and mediastinal involvement is less frequent, about 45% in Maity et al. cohort [16]. Regarding HL features at diagnosis in adults, patients frequently present a localized disease (70 % stage I-II in Casasnovas. et al cohort compared to 55% in our study), whereas the elderly usually present a disseminated but rarely a bulky disease [17, 18].

Additionally, we did not observe any difference in terms of clinical characteristics at diagnosis between patients aged between 15 and 21 years and patients from 21 years, suggesting that the AYA population probably represents a homogeneous population, distinct from children and adults. Our data are in line with the clinical characteristics at diagnosis of the AYA population included in Akhtar et al. cohort, i.e. high rate of disseminated disease (65 %), of mediastinal involvement (70 %), and of extra-nodal involvement (43 %) [19].

Comparing the clinical characteristics at diagnosis between patients diagnosed before and after June 2005, we noticed that the most recent patients tended to present more frequently a disseminated disease. PET-CT might help to detect lesion that cannot been seen by classical CT-scan but it may also lead to false-positive in some cases [20].

As expected, therapeutic management varied over time. Most of the patients were treated according to adult protocols with ABVD and BEACOPP as the two most commonly used regimens. Thus our study confirmed the efficacy of adult regimens in AYAs, which is concordant with the literature [9]. We noticed that MOPP-like treatments were significantly associated with a decreased PFS. It has already been described in the literature that it was less efficient than the ABVD regimen and thus ABVD treatment seems to lead to better PFS [21]. With those different adult regimens, our study did not underline a high rate of treatment- related morbidity or mortality however our median follow-up was only of 6.9 years.

Despite their pejorative baseline features, the prognosis remains excellent in AYA with a 10-year PFS and OS estimated at 81% and 91%, respectively. Stage III and IV according to Ann Arbor, mixed cellularity histology, elevated neutrophils and LDH above range were independently associated with a worse PFS. Although the prognosis remains excellent, we noticed a relapse rate at 16.6%. This rate is consistent with the rate of disseminated HL at diagnosis in this population. Among this population, a small subgroup of patients is distinguishable by a particular aggressive disease and bulky mediastinal involvement which led to resistance to treatment and an extremely pejorative prognosis.

Of note, the salvage chemotherapy and autologous stem cell transplantation does not seem to correct this pejorative prognosis since almost all the refractory patients underwent ASCT and relapsed shortly after. This group of patients should be identified as soon as possible so they could early receive aggressive or new therapeuties such as the antibody- drug conjugate *brentuximab vedotin* and anti PD-L1, which has already shown efficacy in adults and in few cases of pediatric refractory HL [22, 23].

We observed a treatment related toxicity rate below 8%. Solid tumor was the main long-term side effect observed, especially breast cancers. Five patients presented a severe cardiotoxicity which is described in patients treated with old regiments (including a high posology of anthracyclines, and mantle field irradiation at 40/45 Gy). The new regimens such as ABVD should decrease the rate of solid tumors and particularly breast cancers and lead to less cardiotoxicity if the number of courses do not exceed 6, however our follow-up is probably not long enough for the recently treated patients.

Thus, given the excellent results obtained with current standard of care therapies, the challenge is now to develop strategies that aim to reduce acute and long-term toxicity while maintaining high cure rates. It is also urgently needed to better identify patients at high risk of failure requiring early new strategies including targeted therapies.

Our study is inherently limited by its retrospective design and the long period of time can induce heterogeneity due to the change of the standard of care. However, we provide informative insights from a large cohort of 349 patients prospectively enrolled to avoid selection bias.

In conclusion, our study shows that the AYA with HL present pejoratives features, however their prognosis remains good. The adult regimen which is preferentially used in real-life practice seems efficient in this population. Thus, one of the primary goals of the AYA HL’s therapy should be to reduce the risk of long-term toxicity and we are currently evaluating the efficacy and safety of the children protocol OPPA/COPP for girls or OEPA/COPDAC for boys in this specific AYA population [24]. Additionally, we identified primary refractory patients as having a particular pejorative prognosis. These patients present frequently a bulky mediastinum, and the autologous stem cell transplantation is not efficient in such cases. These patients should be identified as soon as possible, in order to propose early new treatment such as anti PD-1 / PDL-1 therapies.

## MATERIALS AND METHODS

### Patients

Consecutive patients diagnosed and treated for HL in the adult onco-hematologic unit of Saint Louis Hospital (AP-HP, Paris, France) from 1979 to 2013 were prospectively recorded in a database along with their baseline characteristics. For follow-up data, we retrospectively reviewed medical charts. Patients with a histological diagnosis of classical HL, regardless of the date of diagnosis, and aged between 15 and 25 years old were included in the study. All diagnoses were based on pathological examination. Immunohistochemistry has been routinely performed since 1995, according to the REAL classification [7]. We collected clinical data such as performance status (according to WHO), stage, histological subtype, biological parameters, treatments and outcome. Patients were treated either in clinical trials, or following available guidelines. Evaluation of response was mainly based on clinical examination, CT scan and PET-CT from 2005. The primary endpoint was progression free survival (PFS), defined as time from first day of treatment until progression, absence of response, relapse, or death from any cause. Primary refractoriness was defined either by progression at any time during chemotherapy or radiotherapy and up to three months after the end of treatment. Early relapse was defined as relapse occurring within the year following the end of the treatment whereas late relapse was defined as relapse which occurs one year after the last treatment [8]. June 2005 was chosen as threshold for the analysis as it constitutes the advent of PET-CT in current practice.

### Treatment

Chemotherapy was given mostly in an outpatient setting and the chemotherapy regimens relied on the HL diagnosis period. As patients were treated in the adult hematologic unit of Saint Louis hospital, almost all the chemotherapy protocols were adult regimens. For patients with the oldest diagnosis, MOPP and MOP/ABV were the most frequently used regimens. ABVD became the standard of treatment from 1986 and escalated BEACOPP has been widely prescribed from 1999 in advanced stages. Adult protocols were mainly ABVD, BEACOPP, MOPP regimen whereas the pediatric protocols were OPPA, OEPA, ABVE-PC.

Radiation therapy (RT) was performed in the same center. The size of the radiation field underwent serial changes during the study period. Initially, extended field RT consisting of mantle field irradiation and/or lumbosacral spleen and/or inverted Y radiation was the standard RT protocol. During the 1990s, the size of the radiation field gradually decreased and involved-field RT was used, which limited treatment to the initially involved nodal areas. Patients were treated with a megavoltage linear accelerator and received a conventionally fractionated RT schedule consisting of 2 Grays (Gy) per fraction in five fractions per week. Doses were progressively reduced during the study period: initially 40/45 Gy, 36 Gy (since 1993), then 30 Gy (since 1998), and more recently 20 Gy (since 2010).

Treatment related mortality was defined as an adverse event probably related to the treatment according to the physician based on clinical features and data of the literature.

### Statistical analysis

Continuous variables were described as median [interquartile range] and categorical variables as numbers (percentage). Marginal association between single variables and the two groups of patients were evaluated using Fisher exact and Wilcoxon sum-rank tests for qualitative and quantitative variables, respectively. PFS was calculated from first day of treatment until progression, relapse, or death from any cause. Time to death was calculated from first day of treatment until death from any cause. Survival functions (PFS and Overall survival, OS) were estimated by the Kaplan-Meier method and compared by the Wald test. Missing data were handled using multiple imputation by chained equation procedure (MICE) considering survival model. Thus, 50 imputed dataset were generated. In order to identify factors associated with the occurrence of an event (progression, relapse, or death from any cause), a univariate Cox proportional hazards regression analysis was performed before and after multiple imputation. Clinical and biological baseline characteristics that were significantly associated with PFS were considered for the multivariate analysis. The final multivariable model was selected using a backward selection method based on the p-values obtained after multiple imputation. Results were reported by hazard ratios (HR) and their 95 percent confidence interval (95%CI). We checked the proportionality of hazard functions for all variables and the log-linearity assumption for continuous variables using restricted cubic regression splines. HRs were compared to 1 using Wald tests. All tests were 2-sided, and p-values less than 0.05 were considered significant. Analyses were performed using R statistical software, version 3.1.3 (http://www.R-project.org).

## SUPPLEMENTARY MATERIALS FIGURES AND TABLES





## Abbreviations

HL: Hodgkin lymphoma
AYA: adolescents and young adults
PFS: progression free survival
OS: overall survival
LDH: lactate dehydrogenase
HIV: human immunodefiency virus
PET-CT: positron emission tomography–computed tomography
HR: hazard ratios
SCT: stem cell transplantation
RT: radiotherapy
CR: complete remission
CRu: uncertain complete remission
