# Supplementary material for: Hodgkin lymphoma in adolescent and young adults: insights from an adult tertiary single-center cohort of 349 patients
Source: Oncotarget. 2017 Sep 6;8(45):80073–82. doi: 10.18632/oncotarget.20684 (PMC5668122; doi:10.18632/oncotarget.20684)
Supplement: Supplementary file 1 [file oncotarget-08-80073-s001.pdf]

Hodgkin lymphoma in adolescent and young adults: insights from an adult tertiary single-center cohort of 349 patients

SUPPLEMENTARY MATERIALS

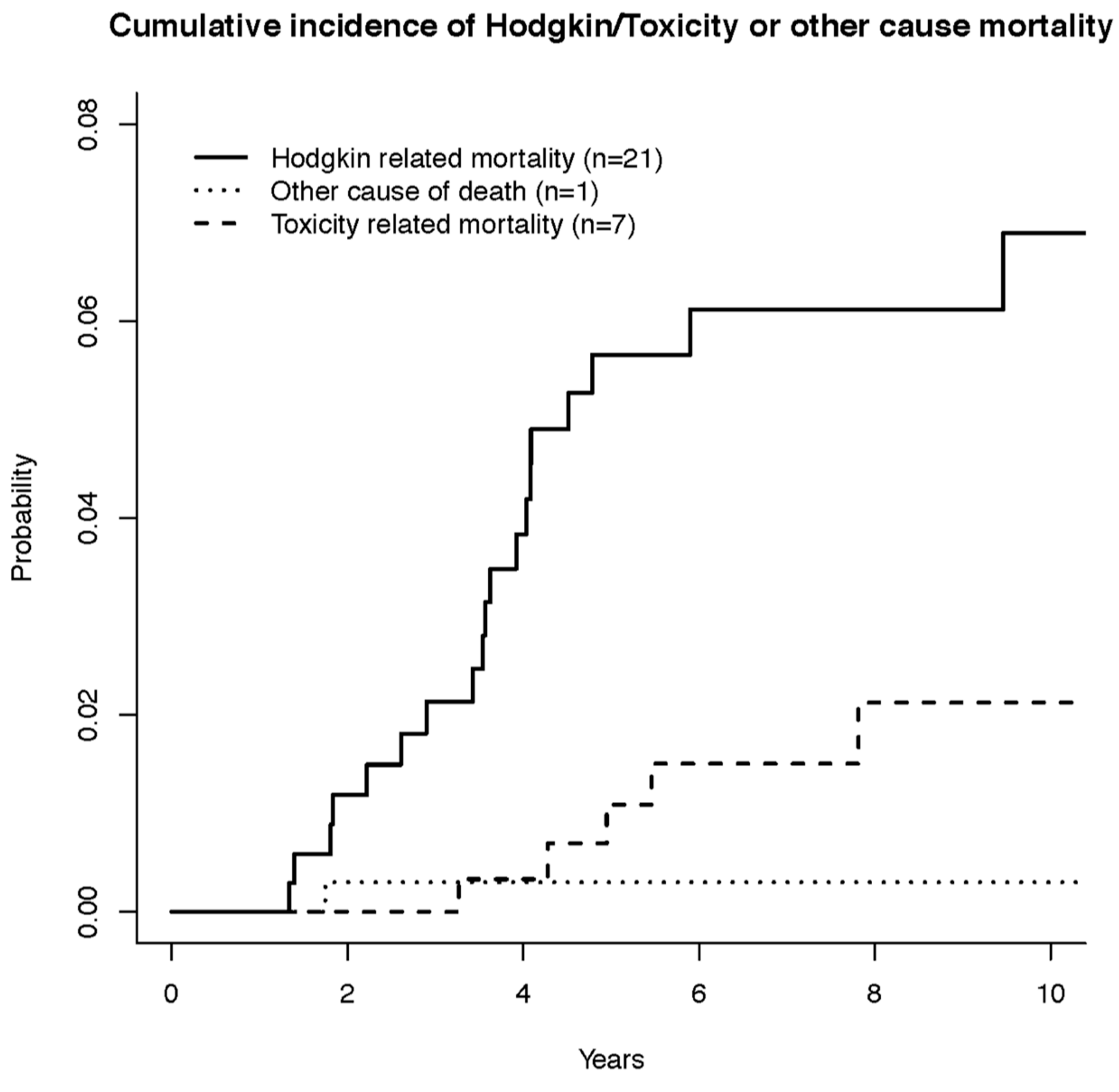

Supplementary Figure 1: Cumulative incidence of overall mortality.

OS according to stage

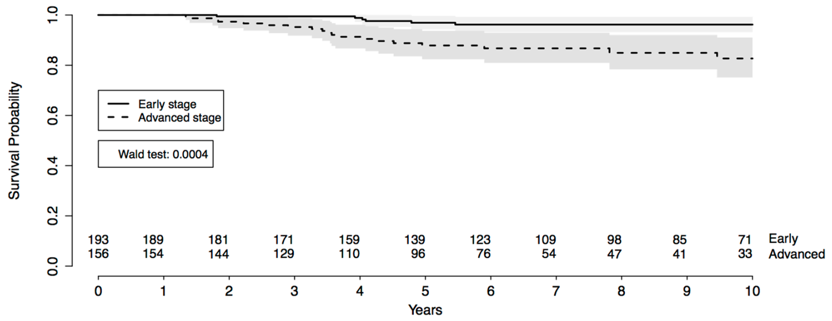

PFS according to stage

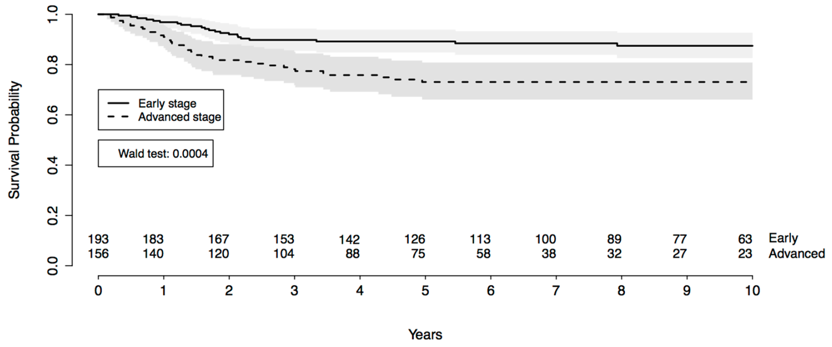

OS according to mediastinal involvement

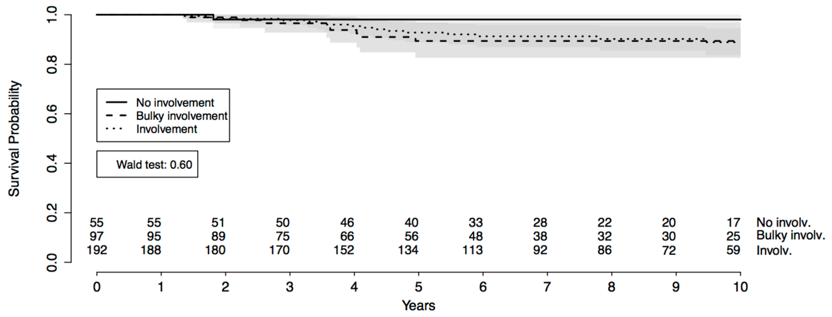

PFS according to mediastinal involvement

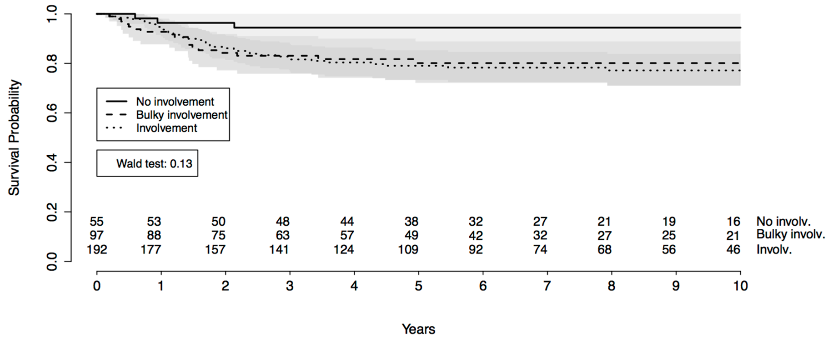

(continued)

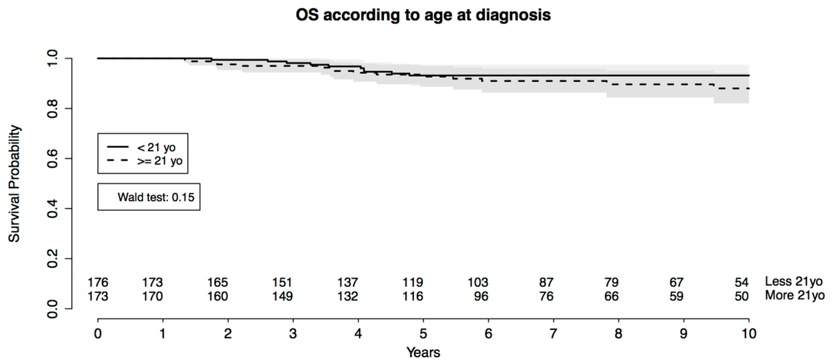
OS according to age at diagnosis
Survival Probability
< 21 yo
>= 21 yo
Wald test: 0.15
176 173 165 151 137 119 103 87 79 67 54 Less 21yo
173 170 160 149 132 116 96 76 66 59 50 More 21yo
Years

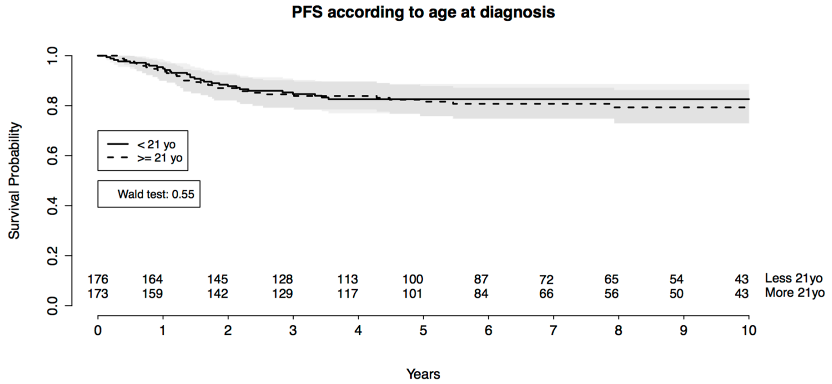
PFS according to age at diagnosis
Survival Probability
< 21 yo
>= 21 yo
Wald test: 0.55
176 164 145 128 113 100 87 72 65 54 43 Less 21yo
173 159 142 129 117 101 84 66 56 50 43 More 21yo
Years

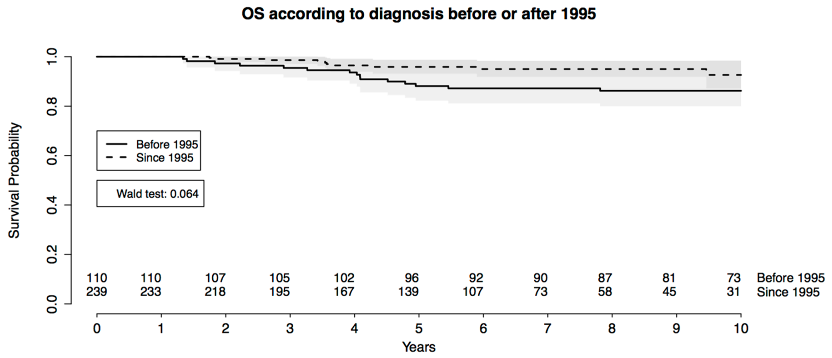
OS according to diagnosis before or after 1995
Survival Probability
Before 1995
Since 1995
Wald test: 0.064
110 110 107 105 102 96 92 90 87 81 73 Before 1995
239 233 218 195 167 139 107 73 58 45 31 Since 1995
Years

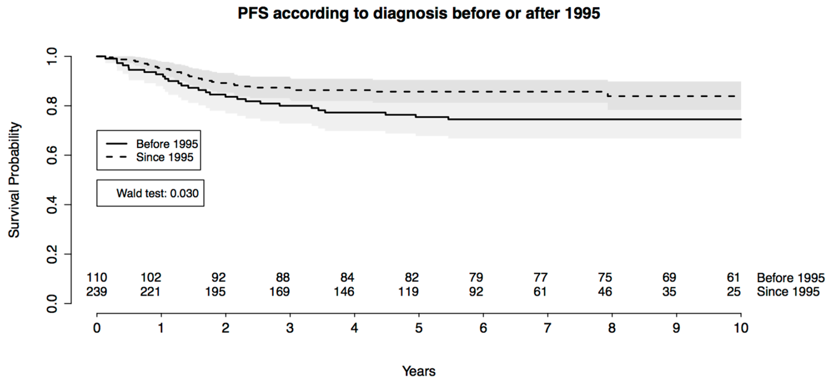
PFS according to diagnosis before or after 1995
Survival Probability
Before 1995
Since 1995
Wald test: 0.030
110 102 92 88 84 82 79 77 75 69 61 Before 1995
239 221 195 169 146 119 92 61 46 35 25 Since 1995
Years

(continued)

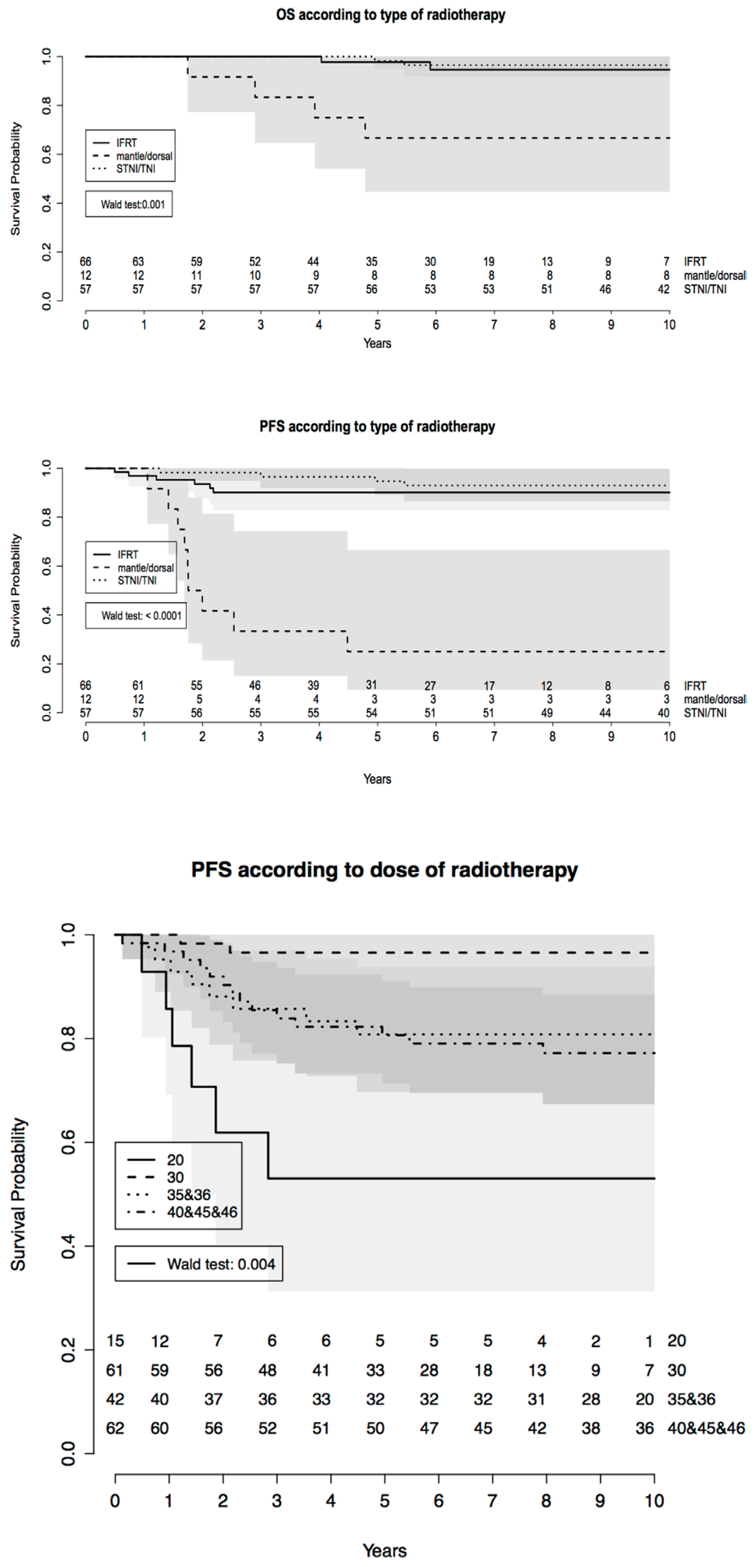

Supplementary Figure 2: Comparisons of 10-year PFS and 10-year OS between patients with early stages vs. advanced stages, bulky vs. non bulky mediastinum, < 21-year old vs. ≥ 21-year old, diagnosis before and after 1995, type of radiotherapy and dose of radiotherapy.

Supplementary Table 1: Comparison between patients less than 21 years-old and patients over 21 years-old

HL characteristics at diagnosis	< 21 year-old (n = 176)	≥ 21 year-old (n=173)	p
**General characteristics**			
Female	100 (56.8 %)	101 (58.4 %)	0.83
Ann Arbor stage			0.83
Stage I and II	96 (54.6 %)	97 (56.1 %)	
Stage III and IV	80 (45.5 %)	76 (43.9 %)	
B symptoms	69 (44.2 %)	80 (47.6 %)	0.58
IPS for stage III-IV	2 [2-3]	2 [2-3]	0.45
Performance status			0.89
0	134 (78.8 %)	135 (79.9 %)	
>=1	36 (21.2 %)	34 (20.1 %)	
Histologic subtype			0.44
Nodular sclerosis	154 (88.0 %)	146 (84.9 %)	
Others	21 (12.0%)	26 (15.1%)	
Negative EBV-LMP staining	70 (89.7 %)	58 (77.3 %)	**0.049**
**Nodal involvement**			
Cervical nodes	152 (87.9 %)	142 (82.6 %)	0.18
Axillary nodes	41 (24.1 %)	34 (20.0 %)	0.43
Mediastinal nodes (including Bulky disease)			0.34
Absent	24 (14.0 %)	31 (18.0 %)	
Present	94 (54.7 %)	98 (57.0 %)	
Bulky disease	54 (31.4 %)	43 (25.0 %)	
Aortic nodes	43 (25.3 %)	41 (24.1 %)	0.90
Iliac nodes	6 (3.5 %)	6 (3.6 %)	1.00
Mesenteric nodes	14 (8.3 %)	12 (7.1 %)	0.84
Pelvic nodes	13 (7.7 %)	14 (8.3 %)	0.84
Spleen	33 (19.4 %)	25 (14.7 %)	0.31
**Extra-nodal involvement**			
Extra-nodal involvement	52 (32.7 %)	63 (38.7 %)	0.30
Lung	32 (18.8 %)	27 (15.9 %)	0.57
Pericardia	12 (7.1 %)	15 (8.9 %)	0.69
Bone	12 (7.5 %)	14 (8.7 %)	0.84
Pleura	11 (6.5 %)	15 (8.8 %)	0.54
Liver	3 (1.8 %)	7 (4.1 %)	0.22
Bone marrow	3 (1.8 %)	4 (2.5 %)	0.72
Oro-pharyngeal	1 (0.6 %)	2 (1.2 %)	1.00
Epiduritis	2 (1.2 %)	1 (0.6 %)	1.00
**Biological characteristics**			
Hemoglobin (g/dl)	12.0 [11.0-13.0]	12.3 [11.1-13.6]	0.22
Leukocytes (G/l)	11.4 [8.3-14.6]	10.3 [8.0-13.5]	0.15
Albumin (g/dl)	40.0 [36.0-43.3]	41.0 [38.0-45.0]	0.19
Lymphocytes (% of white cell count)	15.0 [10.0-21.0]	14.0 [11.0-20.0]	0.53
Neutrophils (% of white cell count)	75 [70.0-80.0]	75 [70.0-80.0]	0.70
Platelets	375.5 [298.5-459.5]	368 [289.3-430.0]	0.82
Lactate dehydrogenase (above normal range)	41 (32.0%)	39 (29.3 %)	0.69
Erythrocyte sedimentation rate (mm/hour)	53.0 [23.5-82.5]	52.0 [27.0-70.8]	0.32

Significant p are depicted in bold.

Supplementary Table 2: Comparison between patients diagnosed before June 2005 and patients diagnosed after June 2005

HL characteristics at diagnosis	Diagnosis before June 2005 (n = 222)	Diagnosis after June 2005 (n = 127)	p value
Histologic subtype, n (%)			**0.02**
Nodular sclerosis	183 (83.2 %)	117 (92.1 %)	
Others	37 (16.8 %)	10 (7.9 %)	
Ann Arbor stage, n (%)			**0.04**
Localized disease (stages I and II)	132 (59.5 %)	61 (48.0 %)	
Disseminated disease (stages III and IV)	90 (40.5 %)	66 (52.0 %)	
Performance status (%)			0.78
0	170 (79.8 %)	99 (78.6 %)	
≥ 1	43 (23.2 %)	27 (21.4 %)	
Cervical node involvement (%)	194 (89.0 %)	100 (78.7 %)	**0.01**
Axillary node involvement (%)	60 (28.2 %)	15 (11.8 %)	**<0.01**
Mediastinal involvement			0.11
Absent	35 (16.1 %)	20 (15.8 %)	
Present	129 (59.5 %)	63 (49.6 %)	
Bulky	53 (24.4 %)	44 (34.7 %)	
Extra nodal involvement, n (%)	68 (34.7 %)	47 (37.3 %)	0.64
LDH (above normal range), n %	54 (32.1 %)	26 (28.0 %)	0.58

LDH: Lactate dehydrogenase.
Significant p are depicted in bold.

Supplementtray Table 3: Academic clinical trials in which patients have enrolled

**Clinical trials**	**References**
LYSA H8 randomized trial	(Fermé *et al*, 2007)
EORTC/GELA H9 randomized trial	(Fermé *et al*, 2017)
LYSA H10 randomized trial	Not yet published
LYSA H89 randomized trial	(Ferme *et al*, 2006)
LYSA H3/4 randomized trial	(Carde *et al*, 2016; Mounier *et al*, 2014)

Supplementary Table 4: First line treatment characteristics

First line treatment	N (%) or median [IQR]	Available for
Chemotherapy		347
ABVD	209 (60.2 %)	
BEACOPP esc	53 (15.2 %)	
MOPP/ABV	39 (11.2 %)	
ABV/IMEP	11 (3.2 %)	
EBVP	11 (3.2 %)	
ABVPP	7 (2 %)	
MOPP	7 (2 %)	
OPPA	1 (0.3 %)	
others	9 (2.6 %)	
Radiotherapy	204 (58.5 %)	349
Type of radiotherapy		204
Involved field radiotherapy (IFRT)	66 (48.9 %)	
Subtotal nodal irradiation (STNI)	52 (38.6 %)	
Mantle field irradiation	11 (8.1 %)	
Total node irradiation (TNI)	5 (3.7 %)	
Dorsal irradiation	1 (0.7 %)	
Unknown	69	
Dose (gray)	36 [30 - 40]	182
Responses		349
Complete response	338 (96.8 %)	
Partial response or progression	11 (3.2 %)	

Supplementary Table 5: Clinical characteristics at relapse/progression and management

Management at first relapse/ progression (n = 58)	N (%) or median [IQR]	Available for
Ann Arbor stage at relapse/progression		45
Stages I and II	27 (60.0 %)	
Stage III and IV	18 (40.0 %)	
Time between relapse and first line treatment		57
median [IQR]	0.86 [0.40 – 1.75]	
< 1 year	35 (60.3 %)	
> 1 year	23 (39.7 %)	
Refractory HL	11 (19.0%)	58
Salvage chemotherapy regimens		57
MINEr	14 (24.6 %)	
IVOx	10 (17.5 %)	
MOPP	6 (10.5 %)	
COP/ABV	5 (8.8 %)	
DHAP	5 (8.8 %)	
ICE	3 (5.3 %)	
others	14 (24.6 %)	
Complementary radiotherapy	10 (17.2 %)	58
Autologous stem cell transplantation (ASCT), n (%)		58
After CR2	37 (63.8 %)	
After late remission	10 (17.2 %)	
Double ASCT	6 (10.3 %)	
Allogeneic stem cell transplantation, n (%)	7 (12.1 %)	58

CR2: complete remission after 2 chemotherapy regimens.

Supplementary Table 6: Multivariate and univariate analysis of prognostic factors for progression free survival

See Supplementary File 1
