# Supplementary material for: Hodgkin lymphoma in adolescent and young adults: insights from an adult tertiary single-center cohort of 349 patients
Source: Oncotarget. 2017 Sep 6;8(45):80073–82. doi: 10.18632/oncotarget.20684 (PMC5668122; doi:10.18632/oncotarget.20684)
Supplement: Supplementary file 2 [file oncotarget-08-80073-s002.docx]

|  |  | **Complete cases univariate analysis** | | | **Univariate analysis after MICE** | | | **Multivariate analysis after MICE** | | |
| --- | --- | --- | --- | --- | --- | --- | --- | --- | --- | --- |
|  |  | **HR** | **IC** | **P value** | **HR** | **IC** | **P value** | **HR** | **IC** | **P value** |
| **Clinical characteristics** | | | | | | | |  |  |  |
| Female |  | 0.68 | [0.41 - 1.11] | 0.12 | 0.68 | [0.41 - 1.11] | 0.12 |  |  |  |
| Age |  | 1.03 | [0.94 - 1.13] | 0.53 | 1.03 | [0.94 - 1.13] | 0.53 |  |  |  |
| Ann Arbor stage | Advanced stage (stage III and VI) | 2.55 | [1.52 - 4.28] | **< 0.01** | 2.55 | [1.52 - 4.28] | **< 0.01** | 2.09 | [1.22 - 3.60] | **< 0.01** |
| International Prognostic Score (IPS) | | 1.28 | [0.94 - 1.73] | 0.12 |  |  |  |  |  |  |
| Performance status different as 0 | | 1.64 | [0.94 - 2.85] | 0.08 | 1.62 | [0.93 - 2.81] | 0.09 |  |  |  |
| Mixed cellularity histology |  | 3.00 | [1.63 - 5.53] | **< 0.01** | 2.95 | [1.60 - 5.45] | **< 0.01** | 2.62 | [1.31 - 5.23] | **< 0.01** |
| Positive EBV-LMP staining |  | 0.83 | [0.29 - 2.41] | 0.73 |  |  |  |  |  |  |
| **Nodal involvement** | | | | | | | |  |  |  |
| Cervical nodes |  | 0.62 | [0.33 - 1.16] | 0.14 | 0.63 | [0.33 - 1.17] | 0.14 |  |  |  |
| Axillary nodes |  | 0.87 | [0.47 - 1.61] | 0.66 | 0.86 | [0.47 - 1.58] | 0.63 |  |  |  |
| Mediastinal nodes |  | 2.55 | [1.02 - 6.37] | **0.045** | 2.61 | [1.05 - 6.53] | **0.04** |  |  |  |
| Aortic nodes |  | 1.48 | [0.86 - 2.56] | 0.16 | 1.49 | [0.88 - 2.55] | 0.14 |  |  |  |
| Iliac nodes |  | 1.59 | [0.50 - 5.08] | 0.43 | 1.24 | [0.40 - 3.88] | 0.71 |  |  |  |
| Mesenteric nodes |  | 1.58 | [0.72 - 3.48] | 0.26 | 1.52 | [0.71 - 3.28] | 0.28 |  |  |  |
| Pelvic nodes |  | 1.06 | [0.42 - 2.64] | 0.91 | 0.93 | [0.38 - 2.29] | 0.88 |  |  |  |
| Spleen |  | 1.61 | [0.88 - 2.92] | 0.12 | 1.58 | [0.87 - 2.88] | 0.13 |  |  |  |
| **Extranodal involvement** | | | | | | | |  |  |  |
| Extra nodal involvement |  | 1.50 | [0.90 - 2.51] | 0.12 | 1.48 | [0.89 - 2.46] | 0.13 |  |  |  |
| Oro-pharyngeal |  | 2.61 | [0.36 - 18.91] | 0.34 | 1.49 | [0.20 - 10.94] | 0.70 |  |  |  |
| Epiduritis |  | 1.93 | [0.27 - 13.98] | 0.52 | 1.46 | [0.22 - 9.83] | 0.70 |  |  |  |
| Lung |  | 2.07 | [1.20 - 3.58] | **<0.01** | 2.05 | [1.19 - 3.55] | **0.01** |  |  |  |
| Pleura |  | 2.33 | [1.15 - 4.73] | **0.02** | 2.14 | [1.05 - 4.36] | **0.04** |  |  |  |
| Pericardia |  | 1.47 | [0.63 - 3.43] | 0.37 | 1.41 | [0.61 - 3.30] | 0.42 |  |  |  |
| Bone |  | 0.70 | [0.22 - 2.24] | 0.55 | 0.88 | [0.31 - 2.48] | 0.80 |  |  |  |
| Bone marrow |  | 1.56 | [0.38 - 6.41] | 0.54 | 1.27 | [0.30 - 5.38] | 0.74 |  |  |  |
| Liver |  | 1.12 | [0.27 - 4.58] | 0.88 | 0.97 | [0.24 - 3.92] | 0.96 |  |  |  |
| **Biological charasteristics** | | | | | | | |  |  |  |
| Hemoglobin (g/dL), effect for 10 units increase | | 0.49 | [0.10 - 2.48] | 0.39 | 0.36 | [0.08 - 1.59] | 0.18 |  |  |  |
| Leukocytes (G/L), effect for 10 units increase | | 2.04 | [1.21 - 3.44] | **< 0.01** | 1.88 | [1.15 - 3.07] | **0.01** |  |  |  |
| Albumin (g/dL), effect for 10 units increase | | 0.49 | [0.30 - 0.80] | **< 0.01** | 0.56 | [0.35 - 0.89] | **0.01** |  |  |  |
| Platelets, effect for 100 units increase | | 1.39 | [1.11 - 1.75] | **< 0.01** | 1.33 | [1.08 - 1.66] | **<0.01** |  |  |  |
| Lymphocytes (% of white cell count), effect for 10 units increase | | 0.52 | [0.31 - 0.86] | **0.01** | 0.58 | [0.36 - 0.94] | **0.03** |  |  |  |
| Neutrophils (% of white cell count), effect for 10 units increase | | 1.97 | [1.24 - 3.12] | **< 0.01** | 1.71 | [1.11 - 2.63] | **0.01** | 1.66 | [1.10 - 2.51] | **0.02** |
| Erythrocyte sedimentation rate, effect for 10 units increase | | 1.09 | [1.00 - 1.18] | **0.045** | 1.08 | [1.00 - 1.17] | **0.049** |  |  |  |
| Lactate dehydrogenase (above normal range) | | 2.29 | [1.24 - 4.23] | **< 0.01** | 2.29 | [1.24 - 4.23] | **< 0.01** | 1.94 | [1.07 - 3.51] | **0.03** |
| **Treatment and date of treatment** | | | | |  |  |  |  |  |  |
| Chemotherapy | ABVD like | 1.00 | Global test : **0.02** | |  |  | |  |  |  |
|  | MOPP like | 2.17 | [1.23 - 3.82] | **<0.01** |  |  |  |  |  |  |
|  | BEACOPP like | 0.61 | [0.24 - 1.56] | 0.31 |  |  |  |  |  |  |
|  | others | 0.53 | [0.07 - 3.88] | 0.54 |  |  |  |  |  |  |
| Radiotherapy |  | 0.64 | [0.39 - 1.06] | 0.09 |  |  |  |  |  |  |
| Type of radiotherapy | IFRT | 1.00 | Global test : **< 0.01** | |  |  | |  |  |  |
|  | STNI + TNI | 0.68 | [0.20 - 2.26] | 0.52 |  |  |  |  |  |  |
|  | Mantle field and dorsal irradiation | 10.51 | [3.69 - 29.95] | **< 0.01** |  |  |  |  |  |  |
| Diagnosis after 1995 |  | 0.57 | [0.35 - 0.95] | **0.03** |  |  |  |  |  |  |
| Diagnosis after june 2005 |  | 0.64 | [0.36 - 1.15] | 0.14 |  |  |  |  |  |  |

HR: Hazard Ratio, CI: Confidence Interval

Significant p are depicted in bold.
